# Temperature-Sensitive Gels for Intratumoral Delivery of **β**-Lapachone: Effect of Cyclodextrins and Ethanol

**DOI:** 10.1100/2012/126723

**Published:** 2012-04-24

**Authors:** Marcilio S. S. Cunha-Filho, Carmen Alvarez-Lorenzo, Ramón Martínez-Pacheco, Mariana Landin

**Affiliations:** ^1^Faculdade de Ciências da Saúde, Universidade de Brasília (UnB), Campus Universitário Darcy Ribeiro, 70910-900 Brasília-DF, Brazil; ^2^Departamento de Farmacia y Tecnología Farmacéutica, Facultad de Farmacia, Campus Vida, Universidad de Santiago, 15782 Santiago de Compostela, Spain

## Abstract

This work evaluated the potential of Pluronics (varieties F127 and P123) in combination with solubilizing agents to be used as syringeable *in situ* gelling depots of intratumoral **β**-lapachone (**β**LAP). Pluronic dispersions prepared at various concentrations in the absence and the presence of ethanol and randomly methylated **β**-cyclodextrin (RM**β**CD) were characterized regarding their rheological properties, drug solubilization capacity, and *in vitro* release. Pluronic F127 (18–23%) formulations combined high ability to solubilize **β**LAP (enhancement solubility factor up to 50), adequate gel temperature range (over 25°C), and gel strength at 37°C enough to guarantee the permanence of the formulation in the administration site for a period of time. **β**LAP release rate was finely tuned by the concentration of the polymer and the addition of RM**β**CD (diffusion coefficient ranging between 9 and 69 **μ**g**·**cm^−2^). The ethanol increases **β**LAP release rate but simultaneously led to weak gels. This paper shows that **β**LAP formulations involving temperature-reversible Pluronic gels may be suitable for intratumoral drug delivery purposes.

## 1. Introduction

Conventional systemic cancer chemotherapy has limited effectiveness in the treatment of solid tumours, the preferred therapy being surgical removal despite frequent recurrence. The use of local controlled release formulations of anticancer drugs is attracting much attention due to the unique physiology of solid tumours, which comprises a highly disordered vasculature and zones of rapidly proliferation cells where a formulation can be retained [1, 2].

*β*-lapachone (*β*LAP) is an anticancer drug that acts by a novel mechanism of direct activation checkpoint regulators inducing apoptosis, particularly useful for neoplasm of slow cell cycle like prostate, pancreatic, colon, and some ovarian and breast cancer [3–6]. Despite its great potential, several factors hinder its use, mainly its low aqueous solubility [7, 8] and its nonspecific distribution which leads to a low tumour concentration and/or a systemic toxicity [9]. An alternative approach for *β*LAP administration could be an injectable formulation containing appropriate polymers able to undergo a transition to semisolid depot into the tumour tissues [1, 10, 11].

Pluronic (poloxamer or Lutrol) amphiphilic triblock copolymers of ethylene oxide (EO) and propylene oxide (PO) blocks (EOx-POy-EOx) self-aggregate as polymeric micelles possessing a relatively hydrophobic core in which poorly soluble drugs can be hosted. The incorporation of the drug into the micelles can improve drug solubility and chemical stability and also regulate the biodistribution and the cell accumulation [12, 13]. Therefore, Pluronic micelles have been shown particularly suitable for the development of formulations of anticancer agents [14] and thus they could be also useful for the photosensitive *β*LAP [15]. Pluronic micellar solutions undergo a sol-to-gel transition when heating above a certain temperature due to a progressive dehydration of PPO and PEO blocks [12]. If the concentration is adequately chosen, the micellar entanglements at body temperature render a 3D physical network that behave as a viscoelastic depot able to sustain drug release at the site of application [10, 16]. Furthermore, pluronics can alter the mechanisms responsible for multidrug-resistance in cancer cells [17, 18]. Both micellization and gelling are reversible phenomena and can be affected by the presence of drugs or cosolvents in the formulation [19]. Nevertheless the findings are sometimes contradictory and, for example, quite opposite results have been reported regarding the effect of ethanol on the critical gel temperature and concentration of Pluronic F127 [20, 21]. The discrepancies could be related to the different technique used to determine the gel point or to the inherent polydispersion of the block copolymers and the presence of some impurities.

The aim of the study was to evaluate the potential of two Pluronic varieties, F127 and P123, to be used in the formulation of temperature-sensitive vehicles of *β*LAP. For an efficient intratumoral delivery of *β*LAP, the following essential criteria should be taken into account: (i) an adequate amount of *β*LAP has to be solubilised; (ii) the formulation should be syringeable in the tumour; (iii) it should form a gel of sufficient consistency to guarantee the formation of a depot system; (iv) the depot should sustain drug release. To carry out the work, aqueous dispersions of the Pluronics at different concentrations were prepared and the effect of additives, such as randomly methylated *β*-cyclodextrin (RM*β*CD) and ethanol, on *β*LAP solubility and gel properties was evaluated. Both RM*β*CD [8] and ethanol could be an aid for drug solubilisation when micelles become saturated. However, both additives could alter the hydrophobic interactions among PPO blocks and thus decrease the solubilisation ability of the micelles [22]. A compromise between both effects should be reached. Additionally, the effect of sterilization by autoclaving on the rheological behaviour and physical stability of the systems was also studied.

## 2. Materials and Methods

### 2.1. Materials

*β*-lapachone (*β*LAP; batch L503; 3,4-dihydro-2,2-dimethyl-2H-naphthol-[1,2-b]pyran-5,6-dione; C_15_H_14_O_3_; MW 242.3) was supplied by Laboratorio Farmacêutico do Estado de Pernambuco, LAFEPE (Recife, Brazil) with purity estimated by DSC and HPLC in 99.9%. Randomly methylated *β*-cyclodextrin (RM*β*CD; degree of substitution 0.57) was kindly donated by Roquette (Barcelona, Spain). Pluronic F127 was purchased from Sigma-Aldrich (St. Louis, USA) and Pluronic P123 was supplied by BASF (Ludwigshafen, Germany). All other chemicals were of analytical grade. Purified water (Millipore, Milli Q Plus, Billerica, USA) was used.

### 2.2. Preparation of Pluronic Dispersions

Stock solutions of Pluronic F127 and P123 were prepared adding the adequate amount of copolymer to water under stirring and kept at 4°C until obtaining a clear transparent solution of 35% (w/w) [12]. Then dilutions of Pluronic F127 (18, 23, or 28% w/w) and P123 (28% w/w) were prepared. When was needed, the drug and the RM*β*CD or ethanol were incorporated in the solution used to dilute the concentrated Pluronic dispersions. Drug concentration tested was 0.2 mg·mL^−1^ and additives concentrations were 5% (w/v) for RM*β*CD and 20% (v/v) for ethanol.

### 2.3. Analytical Methods

*β*LAP was determined spectrophotometrically at 257 nm (Agilent 8453, Germany). Calibration curve in water/ethanol (1 : 1 v/v) was made using standard solutions in the range of 2 to 10 *μ*g^.^mL^−1^. No effect of additives on the spectrum of *β*LAP solution was observed.

### 2.4. Rheological Evaluation

The influence of temperature from 5°C to 40°C on the loss or viscous (G′′) and storage or elastic (G′) moduli of the dispersions was evaluated in a Rheolyst AR-1000 N rheometer (TA Instruments, Newcastle, UK) equipped with an AR2500 data analyzer, a Peltier plate, and a cone with a diameter of 60 mm and 1.58 degree. The gap was fitted to 50 *μ*m. Measurements were performed at 0.5 rad·s^−1^ with an oscillatory stress of 0.1 Pa and a ramp of 2°C·min^−1^. The temperature at which the elastic modulus cross-overs the viscous modulus was considered the gel temperature [19].

Creep-recovery assays were performed at 37°C. During the creep phase, a fixed shear stress of 10 Pa for Pluronic F127 systems and 0.3 Pa for Pluronic P123 systems was applied for 5 min. The samples were equilibrated at 37°C during 2 h before assay. Gel strength was calculated as the inverse of the maximum value of compliance of the retardation phase of creep-recovery profiles. The experiments were carried out in triplicate.

### 2.5. *β*LAP Solubilization

*β*LAP solubility was evaluated by adding an excess of drug into 3 mL of blank Pluronic systems placed in glass ampoules. The suspensions were sonicated during 15 min and mechanically shaken (Gallenkamp, Loughborough UK) at 200 rpm for 7 days at 4°C. Then, the systems were filtered through 0.45 *μ*m nylon filters (Millipore Corp, Billerica, USA) and diluted with water/ethanol solution (1 : 1 v/v) to determine spectrophotometrically *β*LAP concentration. The experiments were carried out in quadruplicate. The enhancement factor (EF) was calculated as the ratio between the *β*LAP solubility value in the system and the *β*LAP solubility in pure water (0.03 mg·mL^−1^) [8].

### 2.6. *In Vitro* Release Assays

*β*LAP diffusion rate from Pluronic dispersions was studied, in quadruplicate, using horizontal side-by-side diffusion cells (Crown glass Corp., Somerville, NJ), separated by a dialysis membrane of a molecular weight cut-off at 7000 Daltons (Visking corp., London, UK) and a diffusional area of 0.64 cm^2^. Pluronic dispersions (2 mL) containing *β*LAP at a concentration of 0.2 mg·mL^−1^ were placed in the donor chamber at 37°C. The receptor medium, phosphate buffer pH 6.8 [23], was continuously stirred with magnetic bar. During 20 h samples were collected regularly and replaced with fresh buffer in order to keep *sink *conditions. The *β*LAP concentration in the solutions was determined as previously described.

### 2.7. Autoclaving

Systems with 28% copolymer containing or not drug and additives were placed in glass ampoules and autoclaved for 20 minutes at 121°C (Raypa model AES-1219, Terrasa, Spain). Samples were then stored at 4°C until characterization. Gel temperature and rheological behaviour after autoclaving were studied as described previously. Samples were observed under optical microscopy (Olympus SZ60 connected to a video camera Olympus DP12, Tokyo, Japan), and the *β*LAP remaining in solution was determined spectrophotometrically.

### 2.8. Statistical Analysis

The statistical analyses of rheological measurements were performed by the one-way analysis of variance (ANOVA) followed by least significant difference test using Statgraphics plus version (*α* = 0.05).

## 3. Results and Discussion

### 3.1. Rheological Properties

The concentration range of Pluronics to produce adequate systems for intratumoral administration was chosen on the basis of preliminary studies. Concentrations between 18–28% for Pluronic F127 and 28% for Pluronic P123 combine syringeability and *in situ* gelling. Below 18% Pluronic F127 does not form gels at 37°C, but above 28%, gels are formed even below 10°C making their handling difficult. The concentration range for obtaining Pluronic P123 systems that undergo the sol-gel transition at body temperature is narrower. Concentrations lower than 28% form gels above 37°C and at a higher concentration the system becomes a gel even at 4°C being not syringeable. The gel temperatures of the systems studied are shown in Table 1. Results of Pluronic F127 systems are in agreement with data in the literature [16]. In the concentration range studied, a close linear correlation between polymer concentration and the gel temperature was observed:

(1) Gel  temperature  (C°)    =  50.67−1.39·(Pluronic  F127  (%));  r2=0.9986.  

The addition of drugs and/or cosolvents can extensively modify the gel temperature of the Pluronic systems [19] with important repercussions on their utility for intratumoral administration. For the Pluronic systems studied, the incorporation of *β*LAP did not cause significant changes (*α* < 0.05) in the gel temperature. By contrast, the addition of ethanol (up to 20%) dramatically decreased the gel temperature of 23% Pluronic F127 and 28% Pluronic P123 estimated as the temperature at which the elastic modulus cross-overs the viscous modulus (Table 1). However, the gels were softer (lower G′ and G′′ after the sol-to-gel transition) than those prepared without additives. Interestingly, the incorporation of ethanol caused 18% Pluronic F127 system to remain as a low viscosity solution at temperatures beyond 37°C, whilst 28% Pluronic F127 gel was not liquefied even below at 4°C. Thus, ethanol caused opposite effects depending on the copolymer concentration. The reasons behind these findings are not clear but should be related to that ethanol makes micellization more difficult, but when micelles are formed, ethanol incorporates into the micellar core causing elongation (worm-like micelles), as already demonstrated for the most hydrophobic variety tested Pluronic P123 [24]. The increase in the micellar size may favour certain entanglement among PEO blocks of neighbour micelles, leading to the cross-over of G′ and G′′ at lower temperature. However, the transition from soft to hard gel requires that the micelles aggregate to reach a percolation threshold. As previously shown using SAXS, disorder in the micellar aggregates results in soft gels, while ordered packing in bcc or fcc structures results in hard gels [21]. Our results are in reasonable agreement with those of Jones et al. [20] and Chaibundit et al. [21] taking into account that the inverted-tube test identifies hard gel formation while the dynamic oscillatory analysis also enables the identification of soft gel formation. Therefore, the addition of ethanol may turn Pluronic dispersions into inappropriate formulations for intratumoral injection purposes. 

The incorporation of RM*β*CD at 5% w/w raised the gel temperature of all systems studied especially of Pluronic P123 dispersions, which is in agreement with results previously reported [25]. This fact can be explained by the formation of supramolecular assemblies named polypseudorotaxanes in which the PPO blocks are inserted in the cyclodextrin cavities [22]. As a consequence, the copolymer unimers involved in the supramolecular structure find that it is difficult to form micelles. Thus, the critical micellar and gelling concentrations of the copolymer increase. 

To gain further insight into the rheological properties of the gels at 37°C, creep-recovery profiles were recorded (Figure 2). The Pluronic dispersions studied can be classified into four groups of different rheological behaviours. Group A, the one with the highest viscosity, includes 23% and 28% Pluronic F127 dispersions without additives or with *β*LAP or *β*LAP and RM*β*CD. Group B corresponds to 18% Pluronic F127 formulated without additives or with *β*LAP or *β*LAP and RM*β*CD. Group C includes the 23% F127 formulations with the drug and ethanol. Group D (with the lowest viscosity) corresponds to 28% Pluronic P123 systems with or without additives. 

The creep-recovery profiles exhibited an important elastic component for groups A and B (Figure 2) with a total recovery around 50%, which means that these systems form a structured network able to store energy. By contrast, groups C and D presented a minor elastic recovery (5%) and a predominant viscous behaviour, which is related to their softer structure. The parameter “gel strength” at 37°C was calculated as the inverse of compliance of the retardation phase of creep-recovery profiles (Table 2). The incorporation of ethanol led to a strong reduction on gel strength and also to a decrease in elasticity for Pluronic F127 dispersions. This confirms that, although gels are formed at lower temperature in the presence of ethanol, the mechanism of gelation and the structure of the network are not the same as in water. The effect of ethanol on Pluronic P123 was negligible. Similarly, cyclodextrin did not significantly alter the viscoelastic profiles at 37°C (Table 2). 

### 3.2. *β*LAP Solubilisation Capacity of the Pluronic Systems

The solubility of *β*LAP in the Pluronic systems and the corresponding enhancement factor (EF) are shown in Table 3. Compared to water, Pluronic F127 formulations notably enhanced *β*LAP solubility up to a copolymer concentration of 23%; beyond that concentration a decrease was observed. General literature indicates that above CMC the solubility of a solute hosted in the micelles should increase linearly with the concentration of the surfactant [26]. The fact that 28% Pluronic solution does not solubilise the drug as well as 23% one does can be related to the relatively high viscosity of 28% Pluronic solutions even at 4°C, which may make drug dissolution and diffusion difficult; the attainment of the equilibrium is delayed. 

In agreement with the HLB values [12], Pluronic P123 (HLB 8) solubilised a higher amount of *β*LAP than Pluronic F127 (HLB 22), although *β*LAP solubility still remained below 1 mg·mL^−1^. For such a poorly soluble drug, the single approach of micellization seems to be not enough to improve the aqueous solubility to the desirable extent [27], its combination with other solubilisation approaches being necessary. Both RM*β*CD (5% w/v) and ethanol (20% v/v) (Table 3) enhanced *β*LAP solubilisation to a great extent. RM*β*CD increased the solubility in almost 50-fold in both Pluronic F127 and P123, reaching drug concentrations close to 1.5 mg·mL^−1^. Ethanol contributed even more to *β*LAP solubilisation, particularly in P123 systems achieving a concentration of 2.4 mg*·*mL^−1^.

### 3.3. *In Vitro* Release Assays

Drug release from an intratumoral implant is mainly controlled by diffusion, since the volume of fluid around the administration site is expected to be small and the dissolution of the polymer and the disintegration of the depot extremely slow [10, 11]. Pluronic systems containing 0.2 mg·mL^−1^ drug concentration with adequate syringeability and gel temperature for intratumoral purposes were tested in regards to their drug release performance using diffusion cells. The selected membrane of 7,000 Da cut-off enabled the movement of the drug towards the receptor compartment, the polymer being retained in the donor compartment. 

**Figure 1:**
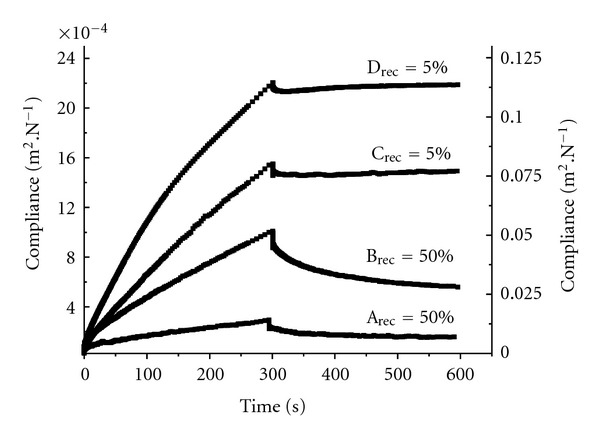
Creep-recovery profiles (37°C) with indications of recovery percentages for the four characteristic rheological behaviour groups, namely, A (left axis): Pluronic F127 at 28% and 23% (without additive; drug; drug + RM*β*CD); B (left axis): Pluronic F127 at 18% (without additive; drug; drug + RM*β*CD); C (left axis): F127 at 23% (drug + ethanol); and D (right axis): Pluronic P123 at 28% (without additive; drug; drug + ethanol; drug + RM*β*CD).

**Figure 2:**
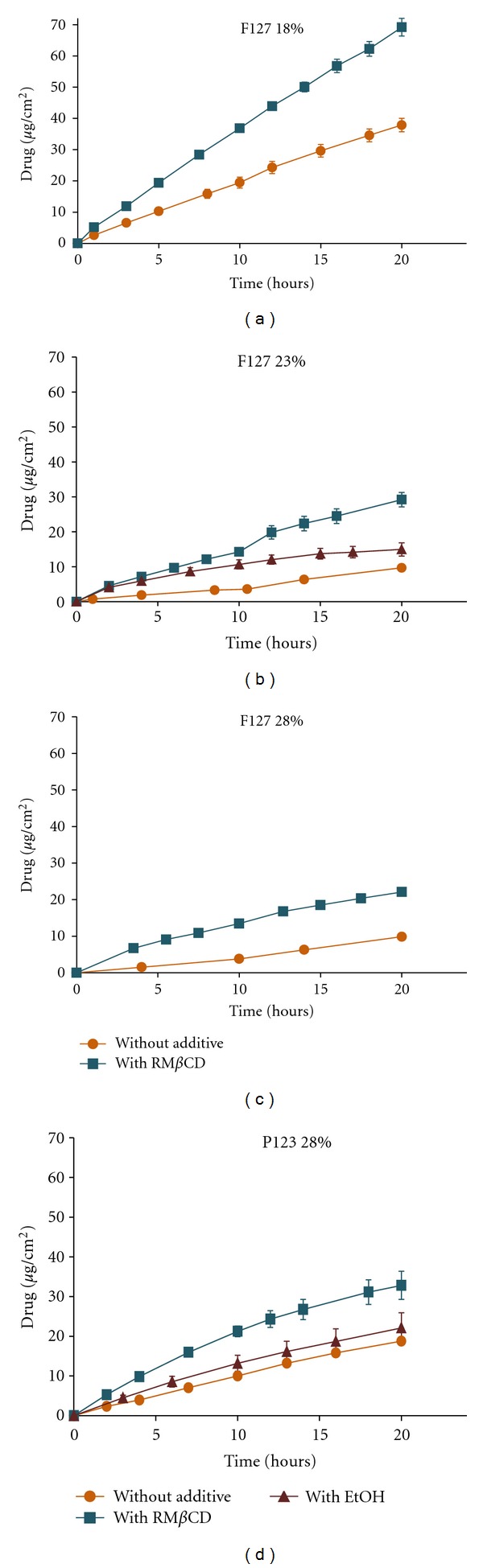
*β*LAP release profiles of Pluronic F127 and P123 systems formulated with and without additives.

As it has been previously described for other drugs, such as sodium diclofenac and quinine formulated in Pluronic gels [28, 29], *β*LAP release profiles fitted well to zero-order kinetics. Formulation including 23% Pluronic F127 and 20% ethanol is the one having the lowest correlation coefficient (*r* = 0.967) but the model was maintained for comparison purposes (Table 4). In this formulation, the presence of ethanol spoils the gel structure of Pluronic F127 affecting its strength (Table 2) and consequently accelerating *β*LAP release rate (Figure 1(b)). By contrast, ethanol does not seem to have any effect on drug release from Pluronic P123 formulation (Figure 1(d)) owing to the less disturbance of the rheological behaviour (Table 2).

A negative correlation was observed between Pluronic F127 concentration and the *β*LAP release rate (Table 4). As the copolymer concentration raises, the entanglement of the copolymer chains also increases limiting drug diffusion [28]. Beyond certain copolymer concentration, the increase in macroviscosity does not correlate well with the hindrance to diffusion and similar release profiles can be obtained [30]. This justifies the similar release kinetics observed for 23% and 28% Pluronic F127 systems. Changes in the release rate of *β*LAP from Pluronic systems containing ethanol could be explained by the reduction in the gel strength of the formulations together with the increment in drug solubility.

Although RM*β*CD did not alter the gel strength profiles of the Pluronic dispersions (Table 2), it greatly accelerated drug release (Figure 1). This effect can be explained by the increase in *β*LAP solubility as a consequence of complex formation with RM*β*CD [8]. The mesh size network of the Pluronic gels seems to be great enough to allow the diffusion of the drug-RM*β*CD complexes.

### 3.4. Autoclaving

**Figure 3:**
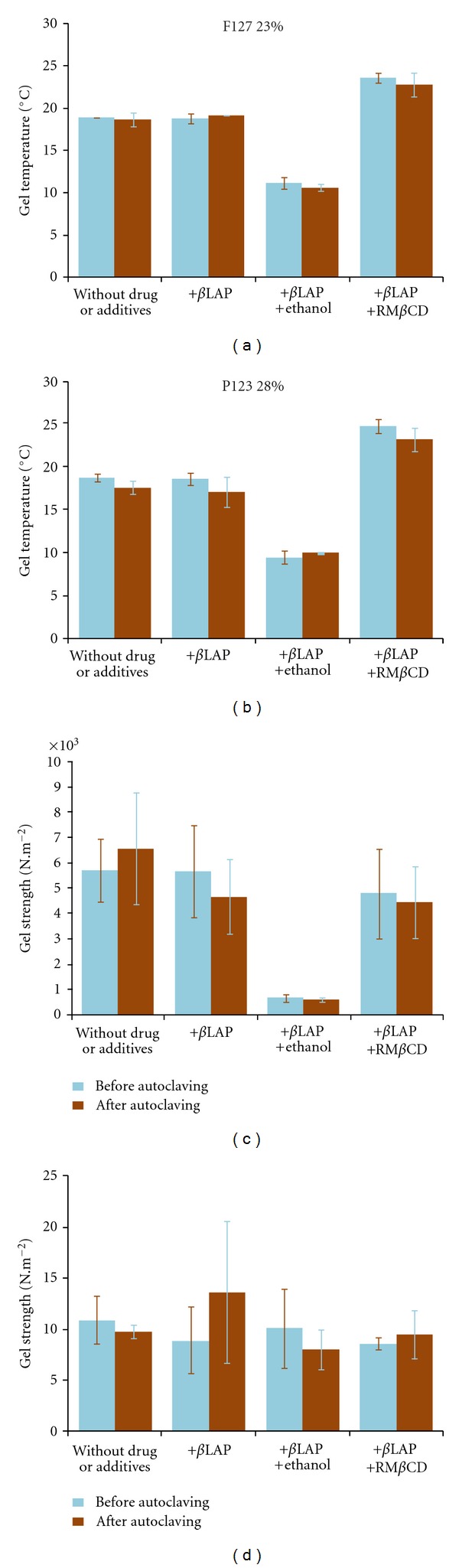
Gel temperature and gel strength at 37°C of Pluronic systems before and after autoclaving.

No statistically significant differences were denoted in the gel temperature or gel strength of the samples before and after autoclaving (Figure 3) which is in agreement with the physical stability of the Pluronic dispersions pointed out by different authors [16, 31]. No crystalline growth was observed under optical microscopy. Samples show that the same UV-visible spectra and *β*LAP amount before and after autoclaving are not significantly different [32]. Therefore, autoclaving could be recommended as a sterilization method for these intratumoral formulations.

## 4. Conclusions

Pluronic P123 systems have a high capacity of *β*LAP incorporation, especially when ethanol (20%) is present in the formulation. However, the low gel strength of those systems does not guarantee the permanence of the formulation in the application site for a long period of time. Pluronic F127 in the 18–23% range presents better perspectives for intratumoral formulation development. It combines adequate gel temperature range (20–30°C) and the gel strength at 37°C may be enough to delay erosion and to control drug release rate. The *β*LAP loading and the release rate can be tuned by the copolymer concentration and the addition of RM*β*CD. The use of ethanol in combination with Pluronic F127 should be avoided as this cosolvent led to soft gels at 37°C. Autoclaving does not affect the physical-chemical properties of the Pluronic systems and may be a suitable sterilization method for the intratumoral formulations. Thus, *β*LAP formulated in temperature-sensitive Pluronic gels may have good perspectives for intratumoral delivery purposes.

## Figures and Tables

**Table 1:** Gel temperature of Pluronic F127 and P123 systems solely or containing 5% (w/v) RM*β*CD and 20% (v/v) ethanol: mean values and, in parenthesis, the standard deviations.

Pluronic %		Without drug or additives	+ *β*LAP	+ *β*LAP+ ethanol	+ *β*LAP+ RM*β*CD
F127	18%	25.5°C (0.0)	24.1°C (0.6)	6.0°C (2.0)	29.1°C (0.7)
	23%	19.0°C (0.0)	18.9°C (0.6)	11.2°C (0.7)	23.7°C (0.6)
	28%	11.6°C (0.6)	12.8°C (0.6)	—	15.7°C (0.8)

P123	28%	18.9°C (0.5)	18.8°C (0.7)	9.6°C (0.8)	25.0°C (0.8)

**Table 2:** Gel strength (N·m^−2^) of the systems at 37°C: mean values and, in parenthesis, the standard deviations.

Pluronic %		Without drug or additives	+ *β*LAP	+ *β*LAP+ ethanol	+ *β*LAP+ RM*β*CD
F127	18%	1554 (542)	1740 (124)	—	1382 (462)
	23%	5740 (1250)	5698 (2821)	692 (156)	4827 (1763)
	28%	6524 (2300)	5174 (583)	—	5892 (850)

P123	28%	10.8 (12.3)	8.8 (3.3)	10.0 (3.9)	8.5 (0.6)

**Table 3:** *β*LAP solubility (mg·mL^−1^) and enhancement factor (EF) in F127 and in P123 systems at 4°C with and without 5% (w/v) RM*β*CD and 20% (v/v) ethanol: mean values and, in parenthesis, the standard deviations.

		Without additives		+ ethanol		+ RM*β*CD	
Pluronic %		[*β*LAP]	EF	[*β*LAP]	EF	[*β*LAP]	EF
F127	18%	0.248 (0.032)	8.3	0.660 (0.051)	22.0	1.037 (0.141)	34.6
	23%	0.444 (0.108)	14.8	1.183 (0.102)	34.4	1.487 (0.117)	49.6
	28%	0.208 (0.028)	7.0	—	—	0.767 (0.141)	25.6

P123	28%	0.875 (0.209)	29.2	2.372 (0.385)	79.1	1.478 (0.383)	49.3

**Table 4:** Data from fitting *β*LAP release profiles to zero-order kinetics and total amount of drug released at the end of the assay (D_20_).

Pluronic %	Additive	Correlation coefficient	Slope (*μ*g h·cm^−2^)	D_20_(*μ*g·cm^−2^)
F127 18%	Without	0.999	1.21	37.9
	5% (w/v) RM*β*CD	0.999	2.19	69.2

F127 23%	Without	0.985	0.30	9.7
	20% (v/v) EtOH	0.967	0.46	14.9
	5% (w/v) RM*β*CD	0.996	0.94	29.2

F127 28%	Without	0.993	0.31	9.9
	5% (w/v) RM*β*CD	0.987	0.68	22.1

P123 28%	Without	0.999	0.61	18.8
	20% (v/v) EtOH	0.994	0.70	22.1
	5% (w/v) RM*β*CD	0.986	1.04	32.8
